# High-Protein Energy-Restriction: Effects on Body Composition, Contractile Properties, Mood, and Sleep in Active Young College Students

**DOI:** 10.3389/fspor.2021.683327

**Published:** 2021-06-15

**Authors:** Christian Roth, Lukas Rettenmaier, Michael Behringer

**Affiliations:** Department of Sports Medicine and Exercise Physiology, Institute of Sport Sciences, Goethe University Frankfurt, Frankfurt, Germany

**Keywords:** fat-free-mass, Tensiomyography, muscle quality, sports nutrition, proteolysis

## Abstract

**Background:** It is often advised to ensure a high-protein intake during energy-restricted diets. However, it is unclear whether a high-protein intake is able to maintain muscle mass and contractility in the absence of resistance training.

**Materials and Methods:** After 1 week of body mass maintenance (45 kcal/kg), 28 male college students not performing resistance training were randomized to either the energy-restricted (ER, 30 kcal/kg, *n* = 14) or the eucaloric control group (CG, 45 kcal/kg, *n* = 14) for 6 weeks. Both groups had their protein intake matched at 2.8 g/kg fat-free-mass and continued their habitual training throughout the study. Body composition was assessed weekly using multifrequency bioelectrical impedance analysis. Contractile properties of the m. rectus femoris were examined with Tensiomyography and MyotonPRO at weeks 1, 3, and 5 along with sleep (PSQI) and mood (POMS).

**Results:** The ER group revealed greater reductions in body mass (Δ −3.22 kg vs. Δ 1.90 kg, *p* < 0.001, partial *η*^2^ = 0.360), lean body mass (Δ −1.49 kg vs. Δ 0.68 kg, *p* < 0.001, partial *η*^2^ = 0.152), body cell mass (Δ −0.85 kg vs. Δ 0.59 kg, *p* < 0.001, partial *η*^2^ = 0.181), intracellular water (Δ −0.58 l vs. Δ 0.55 l, *p* < 0.001, partial *η*^2^ = 0.445) and body fat percentage (Δ −1.74% vs. Δ 1.22%, *p* < 0.001, partial *η*^2^ = 433) compared to the CG. Contractile properties, sleep onset, sleep duration as well as depression, fatigue and hostility did not change (*p* > 0.05). The PSQI score (Δ −1.43 vs. Δ −0.64, *p* = 0.006, partial *η*^2^ = 0.176) and vigor (Δ −2.79 vs. Δ −4.71, *p* = 0.040, partial *η*^2^ = 0.116) decreased significantly in the ER group and the CG, respectively.

**Discussion:** The present data show that a high-protein intake alone was not able to prevent lean mass loss associated with a 6-week moderate energy restriction in college students. Notably, it is unknown whether protein intake at 2.8 g/kg fat-free-mass prevented larger decreases in lean body mass. Muscle contractility was not negatively altered by this form of energy restriction. Sleep quality improved in both groups. Whether these advantages are due to the high-protein intake cannot be clarified and warrants further study. Although vigor was negatively affected in both groups, other mood parameters did not change.

## Introduction

During voluntary weight loss, as much lean body mass as possible should be maintained (Artioli et al., 2010). This, referred to as high-quality weight loss (Churchward-Venne et al., 2013), leads to a better power-to-mass ratio (O'Connor et al., 2007; Turocy et al., 2011), improves efficiency of movement (Sundgot-Borgen and Garthe, 2011), and increases the likelihood of athletic success (Slater et al., 2005; Chappell et al., 2018). However, following low energy availability, muscle protein synthesis is reduced leading to a negative net protein balance, and thus, finally culminates in muscle mass loss (Carbone et al., 2013; Pasiakos et al., 2013). In this context, it has been suggested that higher protein intake (2.4 vs. 1.2 g/kg) might restore muscle protein synthesis (Longland et al., 2016; Macnaughton et al., 2016) due to amino acids being preferentially used for muscle protein synthesis instead of gluconeogenesis (Walberg et al., 1988; Wackerhage and Rennie, 2006), with a concomitant decrease in protein breakdown (Kim et al., 2016; Park et al., 2020). Greater amino acid availability results in a more pronounced positive protein balance (Pikosky et al., 2008; Gwin et al., 2020), leads to a muscle sparing effect and is, therefore, recommended as an efficient strategy to increase the likelihood of lean mass retention (Phillips, 2008, 2014; Manore, 2015; Murphy et al., 2015; Witard et al., 2019).

Various studies examining the impact of energy restriction in active individuals have been conducted (Karila et al., 2008; Pikosky et al., 2008; Morton et al., 2010; Wilson et al., 2012; Pasiakos et al., 2013; Rhyu and Cho, 2014; Huovinen et al., 2015). While most of the studies revealed that energy restriction was associated with a significant lean body mass loss (Karila et al., 2008; Pikosky et al., 2008; Morton et al., 2010; Pasiakos et al., 2013; Rhyu and Cho, 2014), ranging from 34% (~-1200 kcal/day; Morton et al., 2010) to 84% (~-2500 kcal/day; Karila et al., 2008) of the total mass lost per week, some studies reported no significant lean body mass change during energy restriction (Huovinen et al., 2015; Wilson et al., 2015). Since all of these studies differ in total energy deficit, protein intake, sleep duration, baseline body fat, and type of physical activity performed, which are all known to significantly influence lean body mass change (Heymsfield et al., 2011), the exact reasons for the inherent inter-study differences remain unclear. Although higher protein intake during energy deficit may lead to a more favorable lean body mass sparing when compared to lower intakes (Pikosky et al., 2008; Mettler et al., 2010; Wilson et al., 2015; Hudson et al., 2020), it is currently unclear whether a moderately energy-restricted high-protein diet alone is a sufficient stimulus to maintain lean body mass and muscle contractile properties in male college students in the absence of resistance training. Although rapid weight loss procedures have been shown to negatively affect neuromuscular performance (Zubac et al., 2020), a moderate energy restriction may elicit performance-enhancing effects (Pons et al., 2018).

Therefore, the primary aim of this study was to investigate whether a high-protein moderately energy-restricted diet can preserve lean body mass in college students in the absence of resistance training. Furthermore, we investigated if muscle contractility can be preserved during this type of energy restriction. Based on currently available evidence, we hypothesized that a) a high-protein moderately energy-restricted diet is able to preserve the lean body mass even in the absence of resistance training and b) contractile properties are not negatively altered throughout the study. In an attempt to clarify the observed inter-study differences, this study aimed to tightly assess moderator variables affecting lean body mass change (protein intake, sleep duration, body fat, physical activity). Since the majority of previously conducted studies only used pre-post measurements, no precise conclusion can be drawn regarding the time course of lean body mass change. Hence, this study used weekly body composition measurements which have been previously described solely for overweight and obese individuals (Heymsfield et al., 2011).

## Materials and Methods

### Study Design

**Figure 1:**
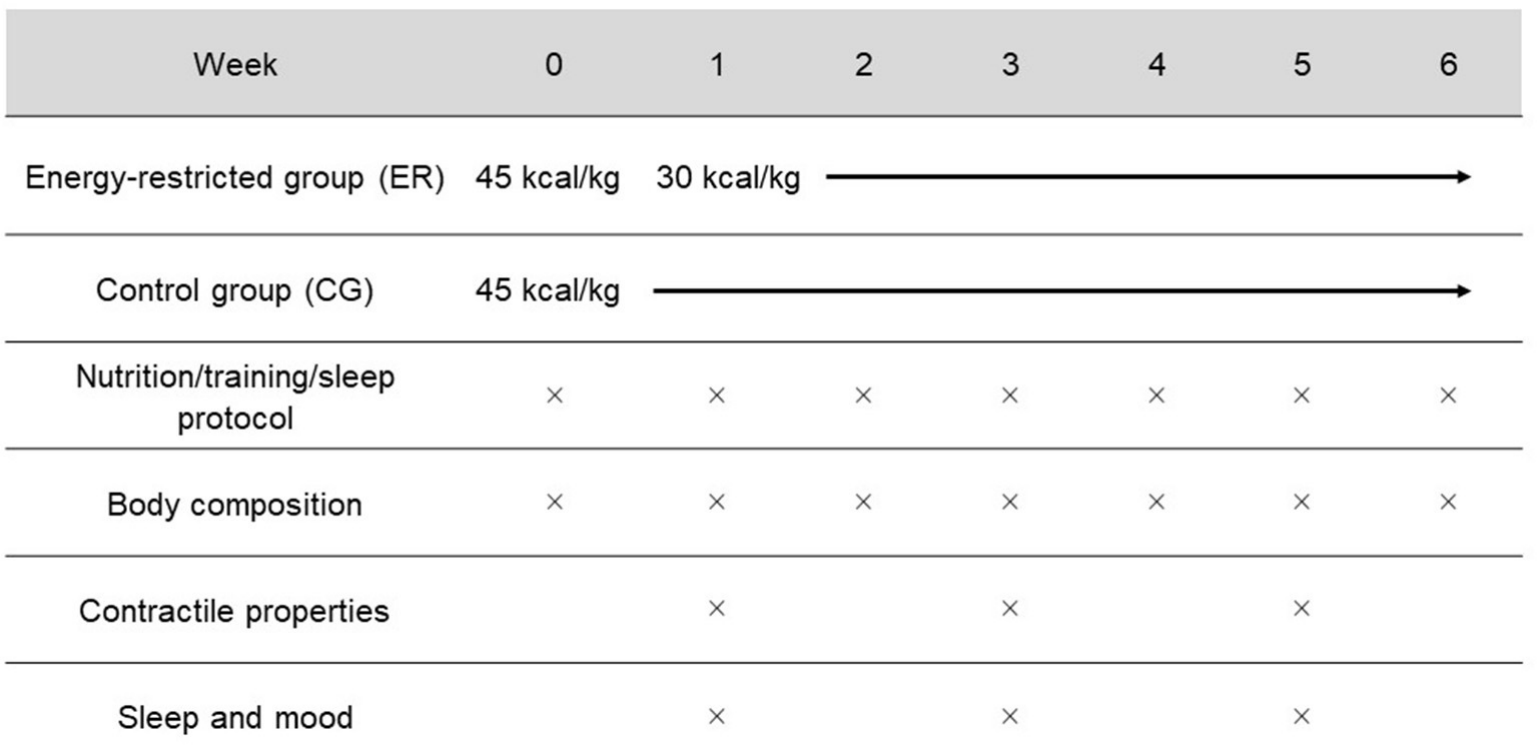
Schematic overview of the study design. In week 0, all participants consumed 100% of their energy requirements (45 kcal/kg). For weeks 1–6, the ER group decreased their energy intake to 30 kcal/kg. Both groups consumed 2.8 g/kg FFM of protein and continued their habitual exercise during the study. As indicated by the × symbol, body composition was assessed weekly. Contractile properties, sleep, and mood were examined at weeks 1, 3, and 5.

The two group, parallel research design was adapted from Mettler et al. (2010) and Philpott et al. (2019) and is illustrated in Figure 1. Once the participants were pair-matched using the variable muscle mass divided by body mass, they were randomly assigned (randomizer.org) to either the energy restriction group (ER, *n* = 14) or the eucaloric control group (CG, *n* = 14). The study protocol consisted of 1 week under eucaloric conditions (45 kcal/kg) for both groups followed by a 6-week intervention period in which the ER group only consumed 30 kcal/kg. The CG maintained their energy intake. Protein consumption was at 2.8 g/kg fat-free-mass (FFM) for both groups during the whole study period. While body composition was assessed weekly via multifrequency bioelectrical impedance analysis (MFBIA), contractile function (Tensiomyography and MyotonPRO), sleep status, and mood were measured in weeks 1, 3, and 5.

The study was approved by the local ethics committee (#2019-24, Goethe University Frankfurt, GER), was conducted in accordance with the ethical standards set by the declaration of Helsinki with its recent modification of Fortaleza (Brazil, October 2013), and met the ethical standards in sport and exercise science according to Harriss and Atkinson (2015). Moreover, the study was preregistered in the International Clinical Trials Registry Platform (WHO) with the registration number DRKS00017263.

### Participants

An *a priori* power analysis was conducted using G^*^Power 3.1 (University Düsseldorf, Germany). The analysis determined that 28 participants were needed for a power of 0.80, with an effect size of *f* = 0.22 and an α = 0.05. Given the fact that lean mass change differs between 0% (Huovinen et al., 2015), 30% (Morton et al., 2010), and up to 84% of the lost mass per week (Karila et al., 2008), no exact effect size calculation was possible. Therefore, we statistically calculated with 30% lean body mass loss and assumed a moderate effect. Accounting for MFBIA precision error and individual variability in lean body mass loss, we further lowered the effect size to detect possible lean mass alterations.

**Figure 2:**
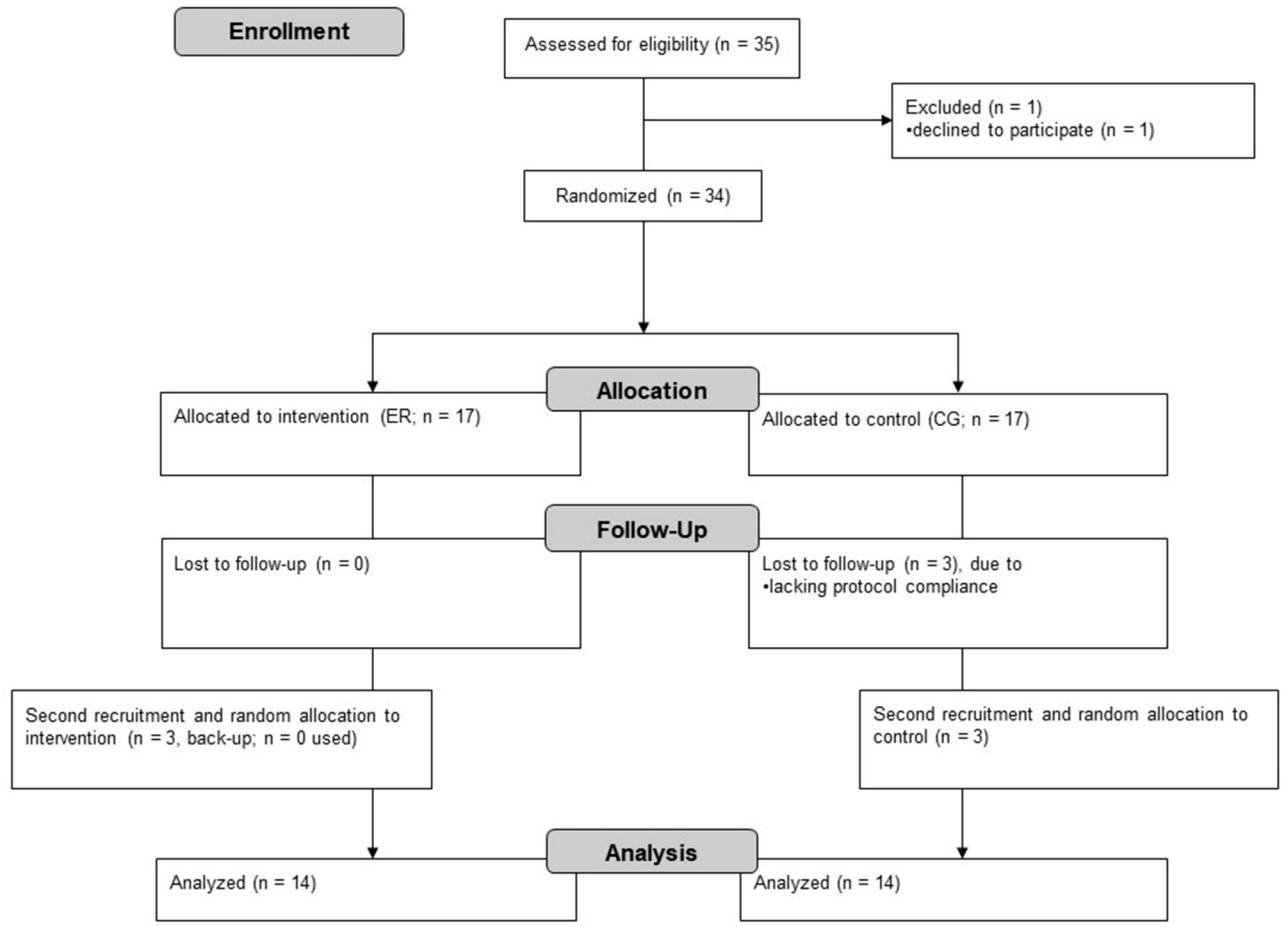
Flow chart of the study conduction following Moher et al. (2001).

Thirty-five healthy males with no experience in resistance training, as assessed by a pre-study questionnaire, were recruited from local sports clubs and university courses (see Figure 2). One participant declined to participate and three participants were excluded due to lacking protocol compliance (did not adhere to dietary intake). Finally, 28 healthy males (ER: age 26.57 ± 4.20 years; height 1.83 ± 0.05 m; body mass 82.26 ± 8.18 kg; CG: age 25.29 ± 2.97 years; height 1.81 ± 0.09 m; body mass 79.19 ± 6.43 kg) were used for data analysis. Due to hormonal fluctuations (Cumberledge et al., 2018), only male participants were included in order to increase reliability. The participants, who all reported that anabolic-androgenic drugs have never been consumed before, undertook at least two sport sessions per week. Since we only aimed for including lean participants, participants were excluded if their body fat was above 25%; this is the cut-off value for obesity, as suggested by Beals et al. (2019). During the study, the participants were asked to continue their habitual training. All participants were informed about the goal of the study as well as its conduction; in particular, interventional strains and requirements were highlighted. Every individual voluntarily agreed and gave written and informed consent to participate in the study.

### Diet and Exercise

On each day during the study, all participants provided self-reported dietary intakes (energy, protein, carbohydrates and fats) using a smartphone app (MyFitnessPal®) as well as their daily body mass. For the latter, participants reported to the nearest 0.1 kg on their own digital scales wearing only underwear. The use of mobile apps for dietary self-reporting is considered to be reliable (Evenepoel et al., 2020). Every subject had either previously used this mobile app or was instructed and taught in a separate one-day workshop given by our lab, as suggested by Capling et al. (2017). In order to increase the compliance rate, the participants received cooking recipes and links to adequate webpages.

In the first week of the study (week 0), both groups had to match an energy intake of 45 kcal/kg. Since our participants reported to be highly active, we decided to stay slightly above the current recommendations of 45 kcal/kg FFM (Economos et al., 1993; Koehler et al., 2016). At the beginning of the intervention period, the ER group decreased their energy to 30 kcal/kg for 6 weeks to induce a moderate energy deficit (Chappell et al., 2018). For data analysis, energy availability was calculated as recommended (Heikura et al., 2018). Protein consumption was controlled during the maintenance and the intervention phase for both groups and was set at 2.8 g/kg FFM (Helms et al., 2014; Hector and Phillips, 2018; Witard et al., 2019). Due to (1) the anabolic effect of protein on muscle protein synthesis, as well as (2) a potential adaptation effect to higher protein intakes with a subsequent increased risk of protein catabolism (Millward, 2001), we aimed for the higher end of the current protein recommendations (Murphy et al., 2015; Bandegan et al., 2017). The remaining energy was individually distributed to carbohydrates and fats as preferred by the participants. Every type of consumed food and drinks (in g and ml respectively) had to be tracked in the nutritional diary. Supplements could be consumed *ad libitum*; however, the participants were asked to abstain from creatine. Compliance was checked weekly by screening all submitted protocols. If unclarities appeared (e.g., protein intake was too low), we kindly asked the participant to improve this issue during the following days. Participants were encouraged to honestly report any non-compliance.

All participants continued their habitual exercise throughout the study. Moreover, the participants provided a self-reported exercise diary on a daily basis as described (Lee et al., 2015). Since resistance training was prohibited during the study, all types of other sports were allowed. The participants were asked to provide sport-specific information for each training session including subjective intensity of the training as well as training duration. Baseline characteristics (week 0) are shown in Table 1.

**Table 1:** Baseline characteristics in the energy-restricted group (ER) and the control group (CG) during the maintenance week (week 0).

	**ER**	**CG**	***p*-value**
Age (years)	26.57 ± 4.20	25.29 ± 2.97	0.358
Height (m)	1.83 ± 0.05	1.81 ± 0.09	0.836
Body mass (kg)	82.24 ± 8.18	79.19 ± 6.43	0.328
BMI (kg/m^2^)	24.68 ± 2.19	24.55 ± 2.54	0.890
Physical activity (minutes/week)	403.27 ± 292.30	389.00 ± 232.03	0.907
Lean mass (kg)	65.87 ± 6.19	64.04 ± 5.36	0.451
Fat mass (%)	20.12 ± 3.90	19.16 ± 3.48	0.534
Energy intake (kcal/day)	3355.88 ± 510.67	3355.61 ± 332.87	0.999
Protein intake (g/day)	182.20 ± 25.55	160.10 ± 22.36	0.036^#^

# *Indicates a significant baseline group difference (p < 0.05) during week 0 as assessed by independent t-test or Mann-Whitney U-test (data in means ± standard deviation)*.

### Measurements

*Body composition* was assessed using MFBIA, 3-compartment model (Nutriguard-MS Vers. 2, Data-Input, Darmstadt, Germany). Examination was conducted as described in the manufacturer's manual following the ESPEN guidelines (Kyle et al., 2004). Briefly, two adhesive electrodes (Bianostic AT, Data-Input, Darmstadt, Germany) were placed on the dominant side of the body: the dorsal surface of the hand and foot proximal to the metacarpal-phalangeal and metatarsal-phalangeal joints. Another two electrodes were placed at the pisiform prominence of the wrist, with the proximal side covering half of the ulnar tubercle, as well as between the medial and lateral malleoli, with the proximal side covering half of the medial malleolus. The dominant side was determined by asking the participants for their dominant side and was maintained for every measurement. In this context, three frequencies (5, 50, and 100 kHz) were used at a current of 800 μA. Uncertainties of resistance (R) and reactance (Xc) given by the manufacturer were depicted as ± 1 ohm and ± 1 ohm, respectively, whilst the precision of measurement was given as 0.5% and 2.0% differing from the value, respectively.

MFBIA (whole body) was tested weekly in a supine position. The same experienced examiner carried out the standardized measurements throughout the entire study period. Participants visited the lab after an overnight fast between 8 and 11 a.m. and emptied their bladder to control for hydration status between the different measurements (Turocy et al., 2011; Bosy-Westphal and Müller, 2014). This was verified by extracellular/intracellular water ratio which is described as a highly sensitive indicator of hydration status change (Wang et al., 2007; Inal et al., 2014; Brzozowska et al., 2019). For instance, deviating toward 1 would suggest a water shift to the extracellular space which is indicative of water loss. Furthermore, the participants were asked to abstain from physical activity the day before testing. Following every testing, a second measurement was conducted to ensure correct values. If the values deviated by more than 3 units digit, a third measurement was conducted and the mean values were calculated. In the context of tracking body composition changes, MFBIA is considered as a reliable tool (Moon, 2013; Bosquet et al., 2017) during hypercaloric (Schoenfeld et al., 2020b) and hypocaloric conditions (Antonio et al., 2019a) in an athletic population, producing similar values as Dual Energy X-ray Absorptiometry (DXA) in males (Golja et al., 2020). Moreover, MFBIA appears to be valid in detecting total body water changes (Utter et al., 2012).

*Tensiomyography* (TMG; TMG-BMC Ltd., Lublijana, Slovenia) was used to assess the contractile function of the m. rectus femoris (dominant side, supine position). TMG is a method to assess radial deformation of the muscle after a single electrical stimulus. Before the first measurement, the center of the anterior inferior iliac spine and the upper edge of the patella was defined, the thickest part of the muscle belly manually palpated and marked with a skin-friendly pen. Subsequently, a high-precision digital displacement sensor was applied perpendicularly to the muscle belly with a spring constant of 0.17 N mm^−1^ (Macgregor et al., 2018) and retracted into its housing by ~2 cm. If necessary, the sensor position was slightly adjusted to locate an area with the greatest amount of muscle belly to sustain an optimal point (Šimunić, 2012). In order to ensure precise inter-day reliability, we strongly encouraged the participants to redraw the marked points following water or sweat-yielding events.

Muscle twitch was induced through a single 1-ms-wide electrical stimulus with the cathode placed distal and symmetrically to the anode (Zubac et al., 2017). The electrodes (self-adhesive; dura-stick plus, 50 × 50 mm), which had an inter-electrode distance of 5 cm as suggested by Piqueras-Sanchiz et al. (2020), were attached on shaved skin. A Blackrole® was deposited under the dominant leg to ensure a leg angle of 120° as suggested (Paula Simola et al., 2015; Sánchez-Sánchez et al., 2018). In order to identify peak muscle response, we progressively increased the intensity at a 10 mA interval every 30 s, beginning with 30 mA (Lohr et al., 2018; Wilson et al., 2018) up until there was no further increase in the amplitude or until maximal output was reached (110 mA) as recommended by Šimunić (2012). Only the curve with the highest maximum of radial displacement was included in the analysis (García-García et al., 2018). In addition to the five standard TMG parameters, which include the maximal radial muscle displacement (D_m_), contraction time (T_c_), delay time (T_d_), sustain time (T_s_), and half relaxation time (Tr), we calculated muscle contraction velocity (V_c_) as D_m_ divided by the sum of T_d_ and T_c_ (Loturco et al., 2016) multiplied by 1000 (mm/s). Relative reliability (ICC) was excellent for D_m_, T_c_, V_c_, and T_d_ during inter-day testing, with T_r_ being the least reliable parameter (Rodriguez Matoso et al., 2010; Šimunić, 2012; Ditroilo et al., 2013; Lohr et al., 2018, 2019).

*MyotonPRO* (MMG; Myoton Ltd., Tallinn, Estonia) was used to extend the muscle quality assessment. In general, MMG is utilized to evaluate viscoelastic characteristics of skeletal muscles and other soft tissues (Aird et al., 2012). MMG causes a light mechanical impulse (0.15 N for 15 ms) to the relaxed muscle and records the natural oscillation of myofascial tissue by a 3-axis digital acceleration sensor sampled at 3200 Hz (Gavronski et al., 2007; Viir et al., 2011). From this raw data, MMG calculates the parameters of stiffness (S, N/m), logarithmic decrement (D, without unit), frequency (F, Hz), relaxation time (R, ms), and creep (C, without unit).

MMG was placed perpendicularly on the same palpated point as described in the TMG section. Per measurement, we applied three measures in multiscan mode, producing five single measures with a 1 s interval. If two of the measures were equal, this value was taken; otherwise, a mean value was calculated. If the coefficient of variation was above 3%, this measure was repeated (Lohr et al., 2018). Most of the studies confirmed good to excellent inter-day reliability for S, D, and F when m. rectus femoris was examined (Bizzini and Mannion, 2003; Zinder and Padua, 2011; Aird et al., 2012; Mullix et al., 2012). Both MMG and TMG were assessed at weeks 1, 3, and 5.

The German version of the *Profile of Mood States* (POMS-G) was utilized to detect possible mood changes during the study period (McNair et al., 1981; Bullinger et al., 1990). A pathopsychological state might affect training performance and, hence, may have an effect on lean mass retention (Franchini et al., 2012; Sundgot-Borgen et al., 2013; Stults-Kolehmainen et al., 2014). Consequently, POMS-G was assessed at weeks 1, 3, and 5. The POMS-G is a frequently-used, reliable and valid questionnaire (Albani et al., 2005; Grulke et al., 2006). In contrast to the original version (McNair et al., 1992), POMS-G is a short form consisting of 35 items and 4 scales (depression-anxiety, fatigue, vigor, and hostility). Each item is assessed on a 7-point Likert scale and retrospectively examines mood state during the last 24 h. Due to its similarities to the English version, our findings can also relate to studies using the English version (Kellmann and Golenia, 2003).

Duration of *sleep* (sleep onset and hours of sleep per night, assessed with a sleep diary) and subjective sleep quality (PSQI-G) were assessed daily and at weeks 1, 3, and 5, respectively. While sleep has mediating effects on testosterone production and muscle protein synthesis (Leproult and van Cauter, 2011; Pejovic et al., 2013), we aimed to clarify the effect of a high-protein energy restriction on sleep quality in healthy male college students. The PSQI is a reliable clinical sleep-behavior questionnaire which was also validated for the general population (Buysse et al., 1989). In contrast to the original version, the PSQI-G assesses the global sleep score in a 2 week interval (Riemann and Backhaus, 1996). The questionnaire contains 19 questions each using Likert scales from 0 to 3 and is categorized into seven sub-variables which are summed up to the PSQI-G score. Regarding cut-off values, scores >5 are associated with a poor sleep condition and ≤ 5 with a good sleep condition (Zhou et al., 2016). During the intervention, we used the standardized procedure as reported (Riemann and Backhaus, 1996).

### Statistical Analysis

A general linear two-way mixed ANOVA with repeated measures [group (2) × time (3/6)] and pairwise comparisons (Bonferroni correction) was performed separately for each dependent variable (SPSS version 24.0, Chicago, IL, USA). When a significant group × time interaction was revealed or the Box's test exposed statistical significance, the simple main effects were examined separately using (a) repeated-measures ANOVA (time) and (b) univariate ANCOVA covarying for t_1_ (group). Before tests were calculated, the research team did an (a) visual review of boxplots, (b) test of normal distribution with the Shapiro-Wilk's test, (c) Levene's test for homogeneity of variance, (d) Box's test of equality of covariance matrices, as well as (e) Mauchly's test of sphericity. Dependent *t*-tests were further carried out to evaluate changes between week 0 and week 1. All tests were based on a 5% level of significance. Data are presented as means ± standard deviation. When possible, effect sizes were reported.

## Results

### Body Composition

A significant group × time interaction was found for body mass [*F*_(3.488,90.676)_ = 14.604, *p* < 0.001, partial *η*^*2*^ = 0.360]. The simple main effect for time revealed a significant body mass loss in the ER group [*F*_(5,65)_ = 12.745, *p* < 0.001, partial *η*^*2*^ = 0.495] between week 1, week 5, and week 6 and a body mass gain in the CG [*F*_(5,65)_ = 6.033, *p* < 0.001, partial *η*^*2*^ = 0.317]. Additionally, significant between-group differences were exhibited beginning at week 2 [*F*_(1,25)_ = 5.156, *p* = 0.032, partial *η*^*2*^ = 0.171]. Consequently, BMI changed significantly from week 1 to week 6 (*p* < 0.001; Table 2).

**Table 2:** Overview of body composition changes in the energy-restricted group (ER) and the control group (CG).

		**Week 0**	**Week 1**	**Week 2**	**Week 3**	**Week 4**	**Week 5**	**Week 6**	**Δ**
Body mass (kg)	ER	82.26 ± 8.18	80.58 ± 8.18^§^	80.14 ± 8.85^#^	79.93 ± 8.47^#^	79.13 ± 8.41^#^	77.92 ± 7.76^#^	77.36 ± 8.00^*^^x^^#^	−3.22
	CG	79.19 ± 6.43	77.49 ± 6.62	78.41 ± 6.34^#^	79.79 ± 6.19^†^^#^	79.37 ± 6.81^#^	79.26 ± 6.58^#^	79.39 ± 6.09^x^^#^	1.90
BMI (kg/m^2^)	ER	24.68 ± 2.19	24.11 ± 2.41					23.13 ± 2.19^x^^#^	−0.98
	CG	24.55 ± 2.54	23.71 ± 2.46					24.29 ± 2.45^x^^#^	0.58
Lean body mass (kg)	ER	65.87 ± 6.19	64.81 ± 5.89^§^	64.82 ± 6.50	64.98 ± 6.18^#^	64.54 ± 5.85^#^	63.69 ± 5.78^#^	63.32 ± 5.84^*^^x^^#^	−1.49
	CG	64.04 ± 5.36	63.33 ± 5.33	63.93 ± 5.31	64.94 ± 5.12^†^^#^	64.46 ± 5.63^#^	64.11 ± 4.96^#^	64.01 ± 5.14^#^	0.68
Body cell mass (kg)	ER	37.92 ± 3.69	37.69 ± 3.80	37.69 ± 4.03^#^	37.89 ± 3.76^#^	37.41 ± 3.71^#^	37.05 ± 3.72^#^	36.84 ± 3.82^*^^#^	−0.85
	CG	36.95 ± 3.44	36.66 ± 3.48	37.26 ± 3.71^#^	37.50 ± 3.44^†^^#^	37.32 ± 3.74^#^	37.19 ± 3.49^#^	37.25 ± 3.74^#^	0.59
Body fat (%)	ER	20.12 ± 3.90	19.44 ± 4.50	18.91 ± 4.56^#^	18.58 ± 4.36^†^^#^	18.24 ± 4.64^#^	17.85 ± 4.39^#^	17.70 ± 4.40^x^^#^	−1.74
	CG	19.16 ± 3.48	17.96 ± 3.90	18.33 ± 3.87^#^	18.72 ± 3.72†^#^	18.74 ± 3.96^#^	19.92 ± 4.14^#^	19.18 ± 3.57^x^^#^	1.22
Intracellular water (l)	ER	28.32 ± 1.94	27.98 ± 1.90^§^	27.91 ± 2.03	28.09 ± 1.94	27.91 ± 1.86	27.65 ± 1.90^#^	27.40 ± 1.96^*^^x^	−0.58
	CG	27.43 ± 2.22	27.25 ± 2.11	27.49 ± 2.20	27.79 ± 2.07	27.86 ± 1.76	27.82 ± 1.65^#^	27.80 ± 1.73	0.55
Extracellular water (l)	ER	19.92 ± 2.68	19.47 ± 2.44	19.49 ± 2.74	19.47 ± 2.60	19.35 ± 2.45	18.99 ± 2.36	18.96 ± 2.38	−0.51
	CG	19.42 ± 3.12	19.11 ± 2.28	19.32 ± 2.19	19.71 ± 2.50	19.32 ± 2.39	19.09 ± 2.04	19.07 ± 2.08	−0.04

§ *Indicates a significant difference between week 0 and week 1 (p < 0.05);*

† *indicates a significant difference between week 1 and week 3 (p < 0.05);*

* *indicates a significant difference between week 3 and week 6 (p < 0.05)*.

x *indicates a significant difference between week 1 and week 6 (p < 0.05)*.

# *indicates a significant between-group difference as shown by the simple main effect for group (p < 0.05); Δ was calculated as week 6–1*.

**Figure 3:**
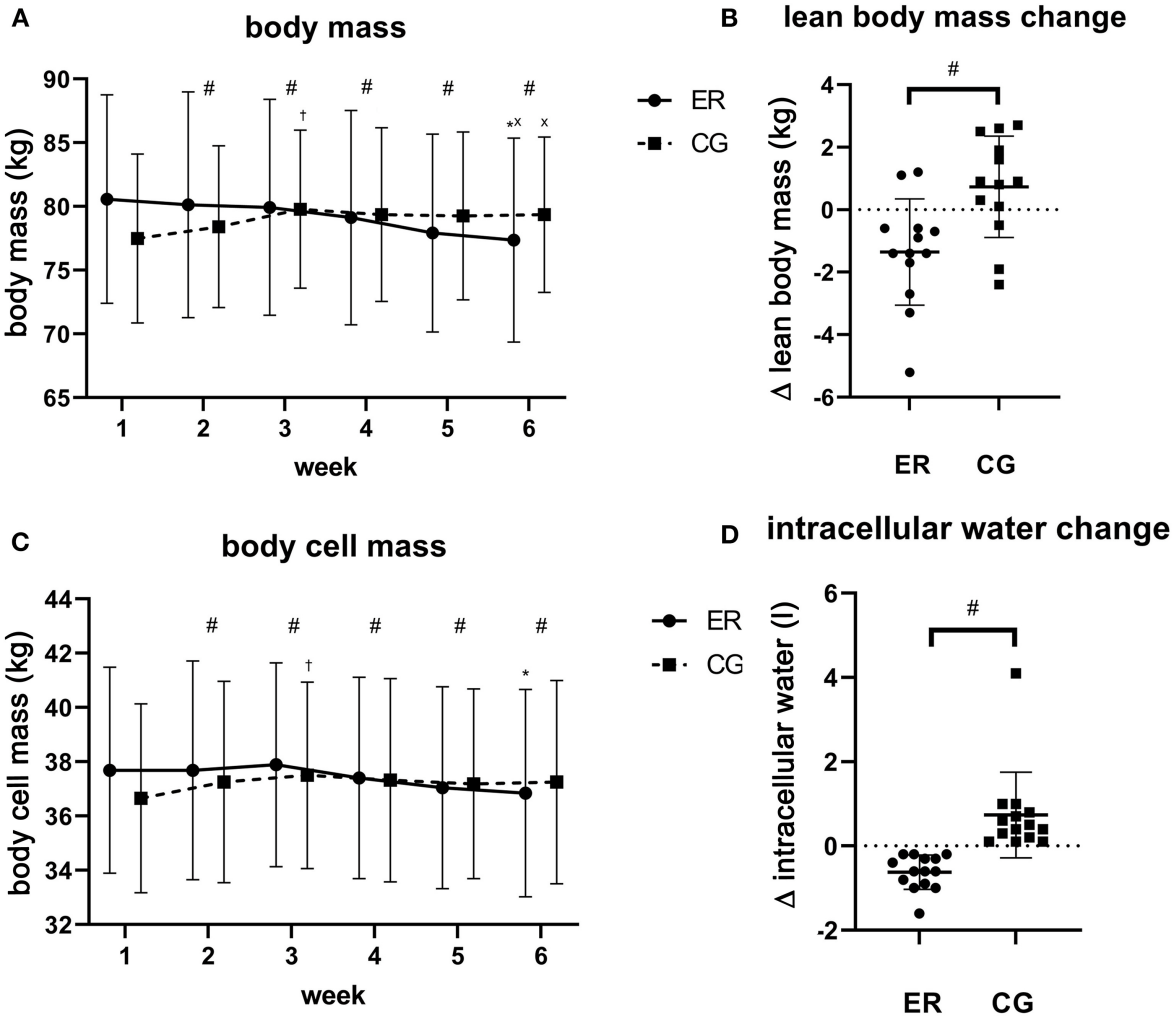
Visual representation of measured parameters. The figure shows body mass change (kg) **(A)**, Δ lean body mass (week 6–1; kg) **(B)**, body cell mass change (kg) **(C)** as well as Δ intracellular water (week 6–1; l) **(D)**. Data is plotted as means ± standard deviation. ^#^Illustrates a significant difference between groups (*p* < 0.05). Indicates a significant difference between week 1 and week 3 (*p* < 0.05); *Indicates a significant difference between week 3 and week 6 (*p* < 0.05). ^x^Indicates a significant difference between week 1 and week 6 (*p* < 0.05). •, energy-restricted group (ER); ■, control group (CG).

A significant group × time interaction was found for lean body mass [*F*_(5,130)_ = 4.673, *p* < 0.001, partial *η*^*2*^ = 0.152; Figure 3]. While lean body mass significantly declined over time in the ER group [*F*_(5,65)_ = 6.181, *p* < 0.001, partial *η*^*2*^ = 0.332], the CG increased lean body mass [*F*_(5,65)_ = 4.369, *p* = 0.002, partial *η*^*2*^ = 0.252]. For the ER group, a significant difference was solely observed between week 3 and week 6 (*p* = 0.002). Contrarily, between-group differences revealed statistical significance at the beginning of week 3 [*F*_(1,25)_ = 6.921, *p* < 0.05, partial *η*^*2*^ = 0.217]. The lean body mass change ranged from +1 kg to −5.2 kg in the ER group and, on average, accounted for 47% of the lost body mass. Hydration status as assessed by extracellular/intracellular water ratio was constant throughout the study in both groups (*p* > 0.05). Further MFBIA derived parameters are collated in Supplementary Table 6.

Similar to what has been reported for lean body mass, the body cell mass, representing the protein-rich and metabolically-active compartments of the body, showed a significant group × time interaction [*F*_(3.190,82.951)_ = 5.740, *p* < 0.001, partial *η*^*2*^ = 0.181]. While the simple main effect for time also exhibited a significant decrease in the ER group [*F*_(5,65)_ = 6.851, *p* = 0.003, partial *η*^*2*^ = 0.345] as well as a significant increase in the CG [*F*_(5,65)_ = 4.078, *p* = 0.003, partial *η*^*2*^ = 0.239], between-group differences were found at the beginning of week 2 [*F*_(1,25)_ = 4.871, *p* < 0.05, partial *η*^*2*^ = 0.163]. Pairwise comparisons over time located the meaningful differences in the ER group between week 3, week 5 and week 6 (*p* < 0.05). While we did not find a group × time interaction for extracellular mass (*p* = 0.10), the main effect for time revealed a change in both groups [*F*_(5,130)_ = 2.592, *p* = 0.029, partial *η*^*2*^ = 0.091]. However, no significant between-group differences were observed for the extracellular mass (*p* = 0.993).

A significant group × time interaction was seen for total body water [*F*_(5,130)_ = 4.681, *p* < 0.001, partial *η*^*2*^ = 0.153]. The simple main effect for time revealed a significant decline in total body water in the ER group [*F*_(5,65)_ = 6.093, *p* < 0.001, partial *η*^*2*^ = 0.319] as well as a significant increase in the CG [*F*_(5,65)_ = 4.259, *p* = 0.002, partial *η*^*2*^ = 0.247], with pairwise comparisons revealing statistical meaningful differences between week 3 and week 6 in the ER group (*p* = 0.003). Moreover, we identified a significant between-group difference for total body water change beginning with week 4 [*F*_(1,25)_ = 4.676, *p* < 0.05, partial *η*^*2*^ = 0.158]. Total body water can be further divided into intracellular and extracellular water. Since both variables revealed a significant Box's test, only the simple main effects were interpreted. While the ER group showed a significant decrease of intracellular water over time [*F*_(5,65)_ = 10.426, *p* < 0.001, partial *η*^*2*^ = 0.445], no change could be detected in the CG (*p* = 0.335). Pairwise comparisons showed significant differences in the ER group between week 1 and week 6, week 2 and week 6, week 3, week 5 and week 6 as well as week 4 and week 6 (*p* < 0.05). Furthermore, significant between-group differences were found at the beginning of week 5 [*F*_(1,25)_ = 5.848, *p* = 0.023, partial *η*^*2*^ = 0.190]. Similar to the extracellular mass, extracellular water decreased only in the ER group [*F*_(5,65)_ = 3.160, *p* = 0.013, partial *η*^*2*^ = 0.196], but did not in the CG (*p* = 0.380). Herein, no between-group differences were observed (*p* > 0.05).

The body fat percentage showed a significant group × time interaction [*F*_(5,130)_ = 19.819, *p* < 0.001, partial *η*^*2*^ = 0.433]. The simple main effect for time exhibited a significant decrease in the ER group [*F*_(2.202,28.623)_ = 14.632, *p* < 0.001, partial *η*^*2*^ = 0.530] as well as a significant increase in the CG [*F*_(2.080,27.036)_ = 6.287, *p* = 0.005, partial *η*^*2*^ = 0.326]. We found a significant difference in the simple main effect for group beginning with week 2 [*F*_(1,25)_ = 11.036, *p* < 0.05, partial *η*^*2*^ = 0.306].

### Diet and Exercise

Food diary analysis showed that the participants in the ER group consumed less energy compared to the maintenance period (*p* < 0.001) and the CG (*p* < 0.001). Regarding energy and protein intake, compliance was >90% on average per group. In individual numbers, the energy intake of the ER group equated to 29.65 ± 1.63 kcal/kg with an energy availability of 31.36 ± 3.13 kcal/kg FFM, respectively (Table 3). In contrast, energy intake of the CG equated to 42.64 ± 2.57 kcal/kg with an energy availability of 48.98 ± 3.36 kcal/kg FFM. Based on Hall's formula (Hall, 2008), the calculated energy deficit was ~-535 kcal/day for the ER group and 316 kcal/day for the CG. Except for week 0, no significant differences were found for protein consumption (*p* > 0.05). Retrospectively, protein consumed was 2.77 ± 0.26 g/kg FFM for the ER group and 2.62 ± 0.33 g/kg FFM for the CG. While the ER group significantly reduced fat and carbohydrate intake between the maintenance and the intervention period (*p* < 0.001), significant between-group differences were spotted in the individual fat (ER: 0.95 ± 0.21 g/kg; CG: 1.45 ± 0.36 g/kg) and carbohydrate (ER: 2.89 ± 0.44 g/kg; CG: 4.79 ± 0.96 g/kg) intake throughout the study period (*p* < 0.001). During the study, the participants in both groups supplemented protein shakes, multivitamin supplements to avoid deficiencies, omega-3 and caffeine.

**Table 3:** Energy intake, dietary intake, and physical activity in the energy-restricted group (ER) and the control group (CG) during the study.

		**Week 0**	**Week 1**	**Week 2**	**Week 3**	**Week 4**	**Week 5**	**Week 6**
Energy (kcal/day)	ER	3355.88 ± 510.67	2396.82 ± 248.86^§^^#^	2386.65 ± 279.88^#^	2362.62 ± 309.36^#^	2372.48 ± 290.45^#^	2351.33 ± 291.52^#^	2382.41 ± 249.81^#^
	CG	3355.61 ± 332.87	3356.78 ± 333.46^#^	3331.54 ± 346.68^#^	3340.70 ± 386.22^#^	3320.97 ± 328.05^#^	3336.28 ± 344.55^#^	3330.19 ± 515.20^#^
Protein (g/day)	ER	182.20 ± 25.55^#^	180.32 ± 24.26	178.59 ± 26.88	179.44 ± 22.16	179.92 ± 20.03	180.09 ± 22.47	190.35 ± 22.75
	CG	160.10 ± 22.36^#^	166.81 ± 26.15	166.78 ± 24.34	168.88 ± 28.89	171.16 ± 25.66	172.19 ± 26.62	160.47 ± 31.99
Fat (g/day)	ER	113.24 ± 25.50	79.22 ± 11.41^§^^#^	75.86 ± 21.37^#^	76.13 ± 23.18^#^	78.30 ± 20.31^#^	71.26 ± 14.92^#^	71.64 ± 15.62^#^
	CG	109.12 ± 29.16	122.63 ± 29.29^#^	119.99 ± 30.15^#^	123.87 ± 38.59^#^	119.04 ± 32.21^#^	122.51 ± 35.93^#^	104.81 ± 34.63^#^
Carbohydrates (g/day)	ER	383.93 ± 92.21	233.77 ± 34.84^§^^#^	239.46 ± 43.76^#^	230.80 ± 44.06^#^	231.31 ± 39.12^#^	236.45 ± 43.20^#^	226.39 ± 33.86^#^
	CG	388.95 ± 88.91	358.33 ± 93.79^#^	367.31 ± 75.96^#^	352.66 ± 84.81^#^	366.58 ± 71.76^#^	366.60 ± 91.18^#^	411.44 ± 77.61^#^
Physical activity (minutes/week)	ER	403.27 ± 292.30	343.08 ± 221.83^§^	294.17 ± 195.68	273.46 ± 175.55	255.83 ± 139.74	367.73 ± 342.91	257.62 ± 143.89
	CG	389.00 ± 232.03	221.43 ± 178.02^§^	268.08 ± 179.66	317.00 ± 195.83	235.83 ± 147.54	234.62 ± 172.22	231.07 ± 135.49

§ *Significantly differed from week 0 (p < 0.05);*

# *indicates a significant between-group difference (p < 0.05)*.

The participants continued their habitual training during the study. In summary, 14 different sports were practiced: gymnastics, bouldering, climbing, soccer, spikeball, bicycling, jogging, table tennis, swimming, volleyball, basketball, boxing, dancing, and paddleboarding. No significant differences in minutes of sport per week, as well as subjective intensity during training were found between the groups (*p* > 0.05). Training sessions per week varied for both groups between 1 and 6 sessions (ER: 4.46 ± 1.76; CG: 2.86 ± 1.29, *p* = 0.012).

### Contractile Properties

**Figure 4:**
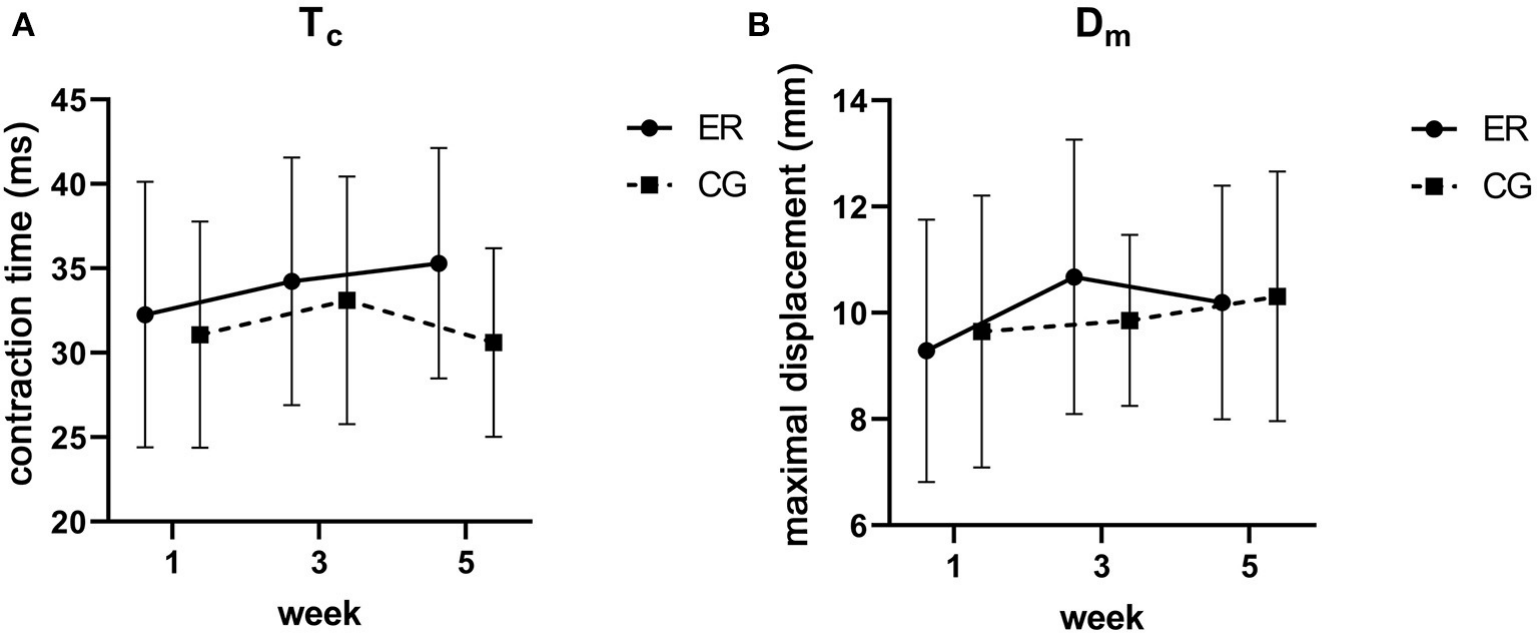
Visual representation of contraction time [T_c_ in ms; **(A)**] and maximal displacement (D_m_ in mm) change **(B)**. Data is plotted as means ± standard deviation. • = energy-restricted group (ER), ■ = control group (CG).

For TMG, no significant differences were found for T_s_ (ER: Δ −4.82 ms; CG: Δ 16.63 ms), T_r_ (ER: Δ −16.65 ms; CG: Δ 16.90 ms) and T_d_ (ER: Δ 1.28 ms; CG: Δ 0.34 ms, all *p* > 0.05). Although group allocation had no effect on T_c_ (ER: Δ 3.04 ms; CG: Δ −0.47 ms), D_m_ (ER: Δ 0.91 mm; CG: Δ 0.66 mm), and V_c_ (ER: Δ 3.84 mm/s; CG: Δ 10.85 mm/s) change, there appears to be an increasing trend in the ER group (*p* = 0.10) as well as in the ER and the CG (*p* = 0.066) for T_c_ and D_m_ over time, respectively (Figure 4; Supplementary Table 7). Lastly, V_c_ significantly increased to week 3 but returned to baseline at week 5.

For MMG, no significant differences were found for stiffness (ER: Δ −4.42 N/m; CG: Δ −4.62 N/m), decrement (ER: Δ −0.04; CG: Δ −0.01), relaxation time (ER: Δ 0.37 ms; CG: Δ 0.08 ms) and creep (ER: Δ 0.02; CG: Δ 0.00, all *p* > 0.05). While frequency did not change over time (ER: Δ 0.00 Hz; CG: Δ 0.04 Hz, *p* > 0.05), the groups differed by trend (*p* = 0.057). An overview of the MMG values is found in Table 4.

**Table 4:** Overview of the MyotonPRO analysis [energy-restricted group (ER), control group (CG)].

		**Week 1**	**Week 3**	**Week 5**
Stiffness (N/m)	ER	246.01 ± 25.20	243.30 ± 23.51	241.59 ± 26.60
	CG	258.70 ± 23.15	252.00 ± 27.69	254.08 ± 27.67
Decrement	ER	1.39 ± 0.19	1.41 ± 0.20	1.35 ± 0.22
	CG	1.42 ± 0.23	1.37 ± 0.23	1.41 ± 0.30
Frequency (Hz)	ER	13.98 ± 0.94	14.07 ± 0.88	13.98 ± 0.86
	CG	14.77 ± 1.11	14.59 ± 1.16	14.81 ± 1.18
Relaxation time (ms)	ER	21.93 ± 1.85	22.12 ± 1.81	22.30 ± 1.71
	CG	21.12 ± 1.59	21.20 ± 1.82	21.20 ± 1.80
Creep	ER	1.34 ± 0.09	1.35 ± 0.10	1.36 ± 0.10
	CG	1.30 ± 0.10	1.30 ± 0.10	1.30 ± 0.10

### Sleep and Mood Analysis

No significant differences were detected for sleep in hours per night and time to fall asleep (*p* > 0.05). While the PSQI-G score significantly decreased over time [*F*_(2,52)_ = 5.568, *p* = 0.006, partial *η*^*2*^ = 0.176], no significant differences were found between the ER group (Δ −1.43) and the CG (Δ −0.64; *p* = 0.247).

Profile of mood states analysis did not reveal a significant difference for depression/anxiety (ER: Δ −2.36; CG: Δ 2.50), fatigue (ER: Δ −3.43; CG: Δ 1.22), and hostility (ER: Δ −3.64; CG: Δ 1.64; all *p* > 0.05). However, vigor decreased significantly over time [*F*_(2,52)_ = 3.417, *p* = 0.040, partial *η*^*2*^ = 0.116] with no differences between the ER group (Δ −2.79) and the CG (Δ −4.71; *p* = 0.583; Table 5). In this context, sleeping hours per night correlated with vigor change (*r* = 0.422, *p* = 0.025).

**Table 5:** Overview of the sleep and mood analysis [energy-restricted group (ER), control group (CG), PSQI-G (Pittsburgh sleep quality index–German)].

		**Week 0**	**Week 1**	**Week 2**	**Week 3**	**Week 4**	**Week 5**	**Week 6**
Sleep per night (hours)	ER	7.42 ± 0.87	7.70 ± 1.02^**§**^	7.71 ± 0.74	7.56 ± 0.63	7.63 ± 0.63	7.74 ± 0.80	7.67 ± 1.15
	CG	7.00 ± 0.96	7.25 ± 0.77	7.37 ± 0.65	7.45 ± 1.01	7.51 ± 0.94	7.38 ± 0.73	7.22 ± 1.30
Time to fall asleep (minutes)	ER	12.54 ± 7.19	13.58 ± 10.92	12.72 ± 9.77	14.46 ± 8.34	14.45 ± 14.89	18.01 ± 21.80	10.65 ± 6.74
	CG	18.89 ± 17.23	12.16 ± 8.36	12.18 ± 8.28	16.66 ± 19.89	11.40 ± 8.32	10.84 ± 6.40	7.05 ± 4.20
PSQI-G-score	ER		5.14 ± 1.75		3.93 ± 0.92		3.71 ± 1.27^*^	
	CG		5.07 ± 2.23		5.14 ± 1.51		4.43 ± 2.03^*^	
**POMS-G**
Depression/anxiety	ER		24.86 ± 9.54		24.86 ± 9.45		22.50 ± 7.52	
	CG		23.86 ± 9.78		27.43 ± 12.43		26.36 ± 13.70	
Fatigue	ER		21.00 ± 6.19		21.21 ± 7.56		17.57 ± 8.67	
	CG		21.14 ± 6.70		20.14 ± 6.89		22.36 ± 8.02	
Vigor	ER		33.00 ± 6.26		31.50 ± 5.49		30.21 ± 8.05^*^	
	CG		32.21 ± 4.89		31.57 ± 7.94		27.50 ± 8.90^*^	
Hostility	ER		14.50 ± 6.98		13.93 ± 8.22		10.86 ± 5.48	
	CG		14.43 ± 6.05		16.79 ± 8.26		16.07 ± 9.47	

§ *Indicates a significant difference between week 0 and week 1 (p < 0.05);*

* *significantly differed to week 1 (p < 0.05)*.

## Discussion

### Body Composition

In this study, we tested the effect of a high-protein moderate energy restriction on body composition change. Generally, maintaining muscle mass is an important health factor due to role of muscle as a primary site of postprandial glucose disposal, lipid oxidation and resting energy expenditure (Hector and Phillips, 2018). In the context of sports, temporary phases of energy restriction are used to reduce body mass while trying to maintain as much lean body mass as possible (Artioli et al., 2010). In particular, lean body mass retention is not only crucial for athletic performance (Wolfe, 2006), but also correlates with athletic success (Slater et al., 2005; Chappell et al., 2018).

In the ER group, lean body mass decreased significantly between week 3 and week 6 with an average total loss of −1.49 kg. According to Siedler et al. (2021), BIA day-to-day variance in lean body mass is as high as 0.9 kg. However, since the decrease in lean body mass is greater than what could be explained by BIA precision error, our data suggest real lean body mass loss in the ER group. With that said, we conclude that the investigated high-protein moderate energy restriction is likely not able to prevent lean mass loss in college students in the absence of resistance training. Consequently, our hypothesis is rejected. Notably, it is unknown whether protein intake at 2.8 g/kg FFM prevented larger decreases in lean body mass. Contrarily, lean body mass was not negatively altered in the CG. Since the CG increased body mass, this indicates a slight caloric surplus.

The energy-restriction-induced reduction of lean body mass is in accordance with the majority of studies (Karila et al., 2008; Pikosky et al., 2008; Morton et al., 2010; Wilson et al., 2012; Pasiakos et al., 2013; Rhyu and Cho, 2014), albeit conflicting results exist (Paoli et al., 2012; Huovinen et al., 2015; Wilson et al., 2015). Since caloric intake, total protein consumption, sex, and sleep duration were taken into account, the inter-study differences may, at least partly, be explained by the magnitude of mechanical tension the body is exposed (Callahan et al., 2021). In one of the studies reporting no significant lean mass change, Paoli et al. (2012) recruited elite artistic gymnasts using a keto-approach (−400 kcal/day, high-protein). With respect to their training regimen, an intense schedule of body weight exercises was carried out which might have led to a greater fiber recruitment of the loaded muscles. In turn, this could have acted as an anabolic stimulus and, in connection with the small energy restriction applied (Karila et al., 2008; Heymsfield et al., 2011), may have led to the retention of muscle mass. This seems to be in accordance with our study showing individual variation in lean mass change in the context of the different types of physical activity performed. Notably, given the fact that Paoli et al. (2012) studied elite athletes, we cannot rule out that strength and conditioning exercises were used additionally without being reported.

Since lean body mass in MFBIA depicts the fat-free compartments of the whole body with muscle mass only representing ~50% (Serra-Prat et al., 2019), solely interpreting the lean body mass change may bias the results. Therefore, body cell mass, representing the protein-rich and metabolically-active compartments of the body (Kyle et al., 2004), i.e., the muscle and organ tissue, is probably the most sensitive marker for muscle loss in MFBIA. In accordance with what has been concluded for the lean body mass change, body cell mass linearly decreased over time in the ER group at the beginning of week 4. In this context, the herein depicted time course of muscle mass loss is in contrast to Heymsfield et al. (2011) who reported an almost linear muscle mass loss at the beginning of the hypocaloric phase in overweight individuals mainly based on the CALERIE study (Heilbronn et al., 2006; Redman et al., 2007; Rickman et al., 2011) and the study by Wood et al. (2007). Contrarily, Schoenfeld et al. (2020a) reported that lean mass loss predominantly occurred during the final weeks of the contest preparation. Since we cannot identify whether these differences might be attributed to the insensitivity of our MFBIA model, other moderator variables, or the potential protective properties of a high-protein dieting approach, this should be studied in future.

### Muscle Contractile Properties

In this study, we tested the effect of a high-protein moderate energy restriction on muscle contractile properties which is, to our knowledge, the first study directly examining the impact of controlled dietary manipulations on TMG and MMG outcomes. In this context, we hypothesized that contractile properties are not negatively altered throughout the study.

Despite depicting high alterations in contractile properties (e.g., muscle force after electrical stimulation of the ulnar nerve) during severe caloric restriction (Lopes et al., 1982; Lennmarken et al., 1986), no group × time interaction was found for any tested variable. With that being said, we conclude that the high-protein moderately energy-restricted diet used in this study did not negatively alter muscle contractile properties. Consequently, our hypothesis is accepted. However, whether this advantage is due to the high-protein diet itself cannot be clarified with the present study and must be examined in future work. Notably, T_c_, which is the contraction time in ms from 10 to 90% of D_m_ on the ascending curve (García-García et al., 2019), tended to increase over time and may reflect a muscle fiber type shift (Valencic and Knez, 1997; Dahmane et al., 2005; Šimunić et al., 2011; Zubac and Šimunić, 2017) in the context of region-specific muscle mass loss (Zubac et al., 2017; Paravlic et al., 2020). However, since different fiber type distributions highly influence the direction of the T_c_ shift (García-García et al., 2013), no exact conclusion can be drawn. Furthermore, a non-significant upwards trend of D_m_ in the ER group (9.8%) was spotted. In this context, D_m_ is seen as an indicator of muscle stiffness whereas a strong negative correlation between D_m_ and stiffness (Macgregor et al., 2018), as well as D_m_ and atrophy (Pišot et al., 2008, 2016) appears to exist. This was expanded by Šimunić et al. (2019) declaring D_m_ as a potential marker of early atrophy. However, since the same non-significant trend, i.e., stiffness loss, was also found in the CG (6.8%), no exact conclusion can be drawn.

Furthermore, the high-protein energy restriction did not show any significant effects on the MMG parameters. However, while the ER group remained at a constant frequency, we noted an upwards trend in the CG. This might reflect higher external loading (e.g., physical activity) since muscle tone amplitude decreases during bed rest (Pišot et al., 2008; Demangel et al., 2017; Schoenrock et al., 2018). Although we cannot rule out that the potential between-group difference is attributed to mechanical tension (Rusu et al., 2013; Schoenrock et al., 2018) or day-to-day variability, physical activity (minutes of sport per week) did not differ between groups. Therefore, we cautiously argue that the greater carbohydrate intake and hence, higher glycogen and intracellular water levels led to a comparably higher muscle tone. As already hypothesized by the following authors (Shiose et al., 2016; Cholewa et al., 2019), carbohydrate loading may increase subcutaneous tension and, thereby, stretches the skin over the evaluated muscle.

### Sleep

Sleep is critical for recovery, performance and lean mass retention (Knufinke et al., 2018; Wang et al., 2018). In our intervention, sleeping hours per night and sleep onset did not change throughout the study as measured by sleep diary. Although diaries might be more accurate than questionnaires, they are prone to recall bias (Halson, 2019) and hence, must be cautiously interpreted. The PSQI-G score, indicative of subjective sleeping quality, decreased significantly in both groups; however, this trend was higher, though not significant, in the ER group compared to the CG. These findings are in contrast to data reported by Driver et al. (1999) who concluded that caloric restriction does not elicit a significant effect on sleep quality in healthy, non-obese men. However, the participants of Driver et al. (1999) only consumed 87 g of protein per day on average and hence, consumed more than 50% less protein compared to our study. The sleep-improving properties of higher protein consumption is described by other authors (Lindseth et al., 2013) and is probably explained by the improved tryptophan to large-chain neural amino acids (Trp-to-LCNAA) ratio. Mediated by a higher insulin secretion, tryptophan is transported across the blood chain barrier and hence, stimulates the synthesis and function of neurotransmitters (e.g., serotonin) as a dietary precursor (Wurtman et al., 2003). However, there seems to be a ceiling effect as seen in athletes who are used to a steady protein supplementation (Antonio et al., 2019b). With that being said, we conclude that a high-protein moderate energy restriction (ER group) may have beneficial effects on sleeping quality which might be greater by trend than a high-protein intake alone (CG). However, due to the lack of low-protein controls, this cannot be clarified and warrants further study.

### Mood

Mood changes are constantly reported in athletic populations (Helms et al., 2019; Reardon et al., 2019). However, there appears to be a plethora of factors influencing mood changes ranging from predisposition, acute biological effects of semi-starvation, to stress due to body monitoring (Helms et al., 2019). Our data predominantly demonstrate no changes in the POMS-G scores. This indicates that neither the moderate energy restriction nor the constant diet, training and body mass tracking had a negative impact on the POMS-G-derived parameters of depression/anxiety, fatigue and hostility. In this context, mood stability might be attributed to the flexible and individual macronutrient profile in our study (Westenhoefer et al., 1999, 2013) and the short duration under energy restriction. Surprisingly, vigor decreased in both groups. In this context, both the ER group and the CG exhibited a significant drop by 10% and 9%, respectively. This is in accordance with most (Degoutte et al., 2006; Koral and Dosseville, 2009; Hulmi et al., 2016), but not all (Wilson et al., 2012) research. For example, in a study by Koral and Dosseville (2009) examining the contest preparation of judokas (−600 kcal/day), the authors reported decreased vigor for the energy restriction but not for the isocaloric controls. Although Koral and Dosseville (2009) attributed the decrease in vigor to body mass loss—supporting the drop revealed in the ER group—this explanation does not fit to the vigor drop shown in the CG in this study. A possible explanation for this might be the high dietary intake that our participants had to consume (45 kcal/kg) of which most of them were not accustomed (Burke et al., 2018).

### Limitations

Nevertheless, our findings need cautious interpretation due to inherent limitations. Overall, the study relied on self-reported dietary intake. Although we controlled total protein intake, meal frequency (Iwao et al., 1996), protein dosage per meal (Loenneke et al., 2016), protein timing (Schoenfeld et al., 2013), and protein source (Gilbert et al., 2011) might also influence lean mass preservation during energy restriction.

In perspective of MFBIA, we found subsequent points worth mentioning. Firstly, hydration status was only assessed using extracellular/intracellular water ratio. Although examination was carried out after an overnight fast, studies intending to replicate our design may use exact measurements of hydration status (e.g., urine-specific gravity) and may also implement a refeeding period after the weight loss intervention to account for possible water fluctuations (Martin-Rincon et al., 2019), as well as their effect on the body cell mass calculation (Walter-Kroker et al., 2011). Secondly, adipose tissue consists of a large extracellular and a small fat-free cell mass per unit weight (Wang and Pierson, 1976; Abe et al., 2019) and, therefore, large amounts of adipose tissue loss may be automatically reported as lean tissue loss. Nevertheless, body cell mass quantification does not take adipocyte changes into account and is likely to be a better marker to decide whether real muscle loss has occurred. Since body cell mass is not only made up of skeletal muscle but also comprises organ tissue, this may also bias interpretation (Nose et al., 1983; Gallagher et al., 2017). Hence, future studies should use DXA or implement a combination of methods (DXA and MFBIA or sonography and BIA/DXA; Haun et al., 2018). Thirdly, regarding the BIA technique, 95% of the impedance is measured in the lower limbs. Thus, the depicted values are mainly derived as a snapshot of lower body changes (Ward, 2019); however, they do seem to be supported by the TMG data.

## Conclusion

In conclusion, the present data show that a high-protein intake alone was not able to prevent lean mass loss associated with a 6-week moderate energy restriction in college students in the absence of resistance training. However, the data revealed that this form of energy restriction did not negatively affect muscle contractility. Sleep quality improved in both groups. This is probably explained by the improved tryptophan to Trp-to-LCNAA ratio; however, there seems to be a ceiling effect as seen in athletes who are used to a steady protein supplementation. Whether these advantages are due to the high-protein intake cannot be clarified due to the lack of low-protein controls and warrants further study. Although vigor was negatively affected in both groups, other mood parameters did not change. In summary, decreasing energy intake moderately while increasing protein consumption does not maintain lean body mass but does maintain contractility in the absence of resistance training in male college students.

## Data Availability Statement

The raw data supporting the conclusions of this article are publicly available. The data can be accessed at the International Clinical Trials Registry Platform (WHO) under the registration number DRKS00017263. Upon request, the data will be made available, without undue reservation.

## Ethics Statement

The study involving human participants was reviewed and approved by the local ethics committee (#2019-24, Goethe University Frankfurt, GER) and was conducted in accordance with the ethical standards set by the declaration of Helsinki. The participants provided their written informed consent to participate in this study.

## Author Contributions

Data analysis was performed by CR and double-checked by LR. Data interpretation was performed by CR, LR, and MB. CR wrote the first draft of the manuscript. All authors contributed to the conception and design of the study, manuscript revision, and read and approved the final version.

## Conflict of Interest

The authors declare that the research was conducted in the absence of any commercial or financial relationships that could be construed as a potential conflict of interest.
