# Corrigendum: High-Protein Energy-Restriction: Effects on Body Composition, Contractile Properties, Mood, and Sleep in Active Young College Students

**DOI:** 10.3389/fspor.2021.762606

**Published:** 2021-11-08

**Authors:** Christian Roth, Lukas Rettenmaier, Michael Behringer

**Affiliations:** Department of Sports Medicine and Exercise Physiology, Institute of Sport Sciences, Goethe University Frankfurt, Frankfurt, Germany

**Keywords:** fat-free-mass, Tensiomyography, muscle quality, sports nutrition, proteolysis

In the original article, there was a mistake in **Supplementary Table 7** as published. There was a computation error in V_c_ week 3, as well as a rounding error in T_r_. The corrected table has been renamed “Supplementary Table 2” and appears below.

**Supplementary Table 2:** Overview of the TMG analysis [energy-restricted (ER) and control group (CG)].

		**Week 1**	**Week 3**	**Week 5**
T_c_ (ms)	*ER*	32.27 ± 7.86	34.24 ± 7.33	35.31 ± 6.83
	*CG*	31.08 ± 6.70	33.12 ± 7.33	30.61 ± 5.82
T_s_ (ms)	*ER*	196.19 ± 64.26	178.37 ± 53.89	191.37 ± 51.65
	*CG*	152.18 ± 53.25	180.11 ± 50.65	168.81 ± 48.87
T_r_ (ms)	*ER*	135.85 ± 68.71	108.56 ± 49.62	119.22 ± 53.04
	*CG*	81.03 ± 49.34	105.10 ± 69.12	97.93 ± 66.99
D_m_ (mm)	*ER*	9.29 ± 2.47	10.68 ± 2.58	10.20 ± 2.20
	*CG*	9.65 ± 2.56	9.86 ± 1.61	10.31 ± 2.35
T_d_ (ms)	*ER*	27.82 ± 3.82	28.54 ± 2.46	29.10 ± 3.66
	*CG*	26.39 ± 2.29	27.19 ± 2.73	26.73 ± 2.36
V_c_ (mm/s)	*ER*	157.67 ± 45.23	173.09 ± 48.70	161.51 ± 43.15
	*CG*	171.67 ± 53.36	167.80 ± 41.74	182.52 ± 48.65

In the original article, there were rounding errors as well as wrongly used signs in Table 2 as published. The corrected Table 2 appears below.

**Table 2:** Overview of body composition changes in the energy-restricted group (ER) and the control group (CG).

		**Week 0**	**Week 1**	**Week 2**	**Week 3**	**Week 4**	**Week 5**	**Week 6**	**Δ**
Body mass (kg)	ER	82.24 ± 8.18	80.58 ± 8.18	80.14 ± 8.85^#^	79.93 ± 8.47^#^	79.13 ± 8.41^#^	77.92 ± 7.76^#^	77.36 ± 8.00^*^^x^^#^	−3.22
	CG	79.19 ± 6.43	77.49 ± 6.62	78.41 ± 6.34^#^	79.79 ± 6.19^†^^#^	79.37 ± 6.81^#^	79.26 ± 6.58^#^	79.39 ± 6.09^x^^#^	1.90
BMI (kg/m^2^)	ER	24.68 ± 2.19	24.11 ± 2.41					23.13 ± 2.19^x^^#^	−0.98
	CG	24.55 ± 2.54	23.71 ± 2.46					24.29 ± 2.45^x^^#^	0.58
Lean body mass (kg)	ER	65.87 ± 6.19	64.81 ± 5.89^§^	64.82 ± 6.50	64.98 ± 6.18^#^	64.54 ± 5.85^#^	63.69 ± 5.78^#^	63.32 ± 5.84^*^^#^	−1.49
	CG	64.04 ± 5.36	63.33 ± 5.33	63.93 ± 5.31	64.94 ± 5.12^†^^#^	64.46 ± 5.63^#^	64.11 ± 4.96^#^	64.01 ± 5.14^#^	0.68
Body cell mass (kg)	ER	37.92 ± 3.69	37.69 ± 3.80	37.69 ± 4.03^#^	37.89 ± 3.76^#^	37.41 ± 3.71^#^	37.05 ± 3.72^#^	36.84 ± 3.82^*^^#^	−0.85
	CG	36.95 ± 3.44	36.66 ± 3.48	37.26 ± 3.71^#^	37.50 ± 3.44^†^^#^	37.32 ± 3.74^#^	37.19 ± 3.49^#^	37.25 ± 3.74^#^	0.59
Body fat (%)	ER	20.12 ± 3.90	19.44 ± 4.50	18.91 ± 4.56^#^	18.58 ± 4.36^†^^#^	18.24 ± 4.64^#^	17.85 ± 4.39^#^	17.70 ± 4.40^x^^#^	−1.74
	CG	19.16 ± 3.48	17.96 ± 3.90	18.33 ± 3.87^#^	18.72 ± 3.72^†^^#^	18.74 ± 3.96^#^	18.92 ± 4.14^#^	19.18 ± 3.57^x^^#^	1.22
Intracellular water (l)	ER	28.32 ± 1.94	27.98 ± 1.90^§^	27.91 ± 2.03	28.09 ± 1.94	27.91 ± 1.86	27.65 ± 1.90^#^	27.40 ± 1.96^*^^x^^#^	−0.58
	CG	27.43 ± 2.22	27.25 ± 2.11	27.49 ± 2.20	27.79 ± 2.07	27.86 ± 1.76	27.82 ± 1.65^#^	27.80 ± 1.73^#^	0.55
Extracellular water (l)	ER	19.92 ± 2.68	19.47 ± 2.44	19.49 ± 2.74	19.47 ± 2.60	19.35 ± 2.45	18.99 ± 2.36	18.96 ± 2.38	−0.51
	CG	19.42 ± 3.12	19.11 ± 2.28	19.32 ± 2.19	19.71 ± 2.50	19.32 ± 2.39	19.09 ± 2.04	19.07 ± 2.08	−0.04

In the original article, there was a mistake in the legend for **Supplementary Table 7** as published. Since the V_c_ values for week 3 were corrected due to a computation error, the sign for significance is no longer needed. The name of the table has been corrected to “Supplementary Table 2” and the corrected legend appears below.

Supplementary Table 2 Overview of the TMG analysis [energy-restricted (ER) and control group (CG)].

In the original article, the computation error in V_c_ as well as a rounding error in T_r_ have been corrected.

A correction has been made to **Results**, Contractile Properties, Paragraph 1:

For TMG, no significant differences were found for T_s_ (ER: Δ −4.82 ms; CG: Δ 16.63 ms), T_r_ (ER: Δ −16.63 ms; CG: Δ 16.90 ms) and T_d_ (ER: Δ 1.28 ms; CG: Δ 0.34 ms, all *p* > 0.05). Although group allocation had no effect on T_c_ (ER: Δ 3.04 ms; CG: Δ −0.47 ms), D_m_ (ER: Δ 0.91 mm; CG: Δ 0.66 mm), and V_c_ (ER: Δ 3.84 mm/s; CG: Δ 10.85 mm/s) change, there appears to be an increasing trend in the ER group (*p* = 0.10) as well as in the ER and the CG (*p* = 0.066) for T_c_ and D_m_ over time, respectively (Figure 4; **Supplementary Table 2**).

In the original article, there was a rounding error in the body mass (ER group). A correction has been made to **Materials and Methods**, Participants, Paragraph Number 2:

Thirty-five healthy males with no experience in resistance training, as assessed by a pre-study questionnaire, were recruited from local sports clubs and University courses (see **Figure 2**). One participant declined to participate and three participants were excluded due to lacking protocol compliance (did not adhere to dietary intake). Finally, 28 healthy males (ER: age 26.57 ± 4.20 years; height 1.83 ± 0.05 m; body mass 82.24 ± 8.18 kg; CG: age 25.29 ± 2.97 years; height 1.81 ± 0.09 m; body mass 79.19 ± 6.43 kg) were used for data analysis. Due to hormonal fluctuations (Cumberledge et al., 2018), only male participants were included in order to increase reliability. The participants, who all reported that anabolic-androgenic drugs have never been consumed before, undertook at least two sport sessions per week. Since we only aimed for including lean participants, participants were excluded if their body fat was above 25%; this is the cut-off value for obesity, as suggested by Beals et al. (2019). During the study, the participants were asked to continue their habitual training. All participants were informed about the goal of the study as well as its conduction; in particular, interventional strains and requirements were highlighted. Every individual voluntarily agreed and gave written and informed consent to participate in the study.

The authors apologize for this error and state that this does not change the scientific conclusions of the article in any way. The original article has been updated.

## Publisher's Note

All claims expressed in this article are solely those of the authors and do not necessarily represent those of their affiliated organizations, or those of the publisher, the editors and the reviewers. Any product that may be evaluated in this article, or claim that may be made by its manufacturer, is not guaranteed or endorsed by the publisher.
