# Supplementary material for: High-Protein Energy-Restriction: Effects on Body Composition, Contractile Properties, Mood, and Sleep in Active Young College Students
Source: Front Sports Act Living. 2021 Jun 15;3:683327. doi: 10.3389/fspor.2021.683327 (PMC8239143; doi:10.3389/fspor.2021.683327)
Supplement: Supplementary file 1 [file Table_1.DOCX]

Supplementary Material

**Table 6.** Further MFBIA variables (energy-restricted (ER) and control group (CG)). ^§^ indicates a significant difference between week 0 and week 1; ^†^ indicates a significant difference between week 1 and week 3_;_ * indicates a significant difference between week 3 and week6. ^x^ indicates a significant difference between week 1 and week 6. ^#^ indicates a significant between-group difference as shown by the simple main effect for group

|  |  | **week 0** | **week 1** | **week 2** | **week 3** | **week 4** | **week 5** | **week 6** |
| --- | --- | --- | --- | --- | --- | --- | --- | --- |
| **Phase angle (°)** | ER | 7.30 ± .57 | 7.46 ± .44^§^ | 7.44 ± .47 | 7.49 ± .45 | 7.39 ± .48 | 7.44 ± .50 | 7.44 ± .45 |
|  | CG | 7.34 ± .54 | 7.38 ± .56 | 7.47 ± .57 | 7.36 ± .58 | 7.36 ± .57 | 7.40 ± .55 | 7.41 ± .58 |
| **Total Body Water (l)** | ER | 48.22 ± 4.53 | 47.45 ± 4.32^§^ | 47.44 ± 4.76 | 47.55 ± 4.53 | 47.24 ± 4.27^#^ | 46.64 ± 4.23^#^ | 46.34 ± 4.27*^#^ |
|  | CG | 46.88 ± 3.92 | 46.36 ± 3.91 | 46.79 ± 3.89 | 47.53 ± 3.75^†^ | 47.17 ± 4.10^#^ | 46.91 ± 3.63^#^ | 46.88 ± 3.77^#^ |
| **Extracellular Mass (kg)** | ER | 27.95 ± 3.16 | 27.06 ± 2.58^§^ | 27.12 ± 2.89 | 27.08 ± 2.77 | 27.12 ± 2.62 | 26.65 ± 2.57 | 26.57 ± 2.40^x^ |
|  | CG | 27.07 ± 2.61 | 26.66 ± 2.54 | 26.66 ± 2.27 | 27.42 ± 2.41 | 27.14 ± 2.56 | 26.93 ± 2.15 | 26.85 ± 2.22^x^ |
| **ECW/ICW ratio** | ER | .70 ± .05 | .69 ± .04 | .70 ± .05 | .69 ± .05 | .69 ± .05 | .68 ± .04 | .72 ± .05 |
|  | CG | .71 ± .13 | .70 ± .08 | .70 ± .08 | .71 ± .10 | .69 ± .05 | .68 ± .04 | .72 ± .07 |
| **Body fat (kg)** | ER | 16.37 ± 4.00 | 15.76 ± 4.44 | 15.32 ± 4.63^#^ | 14.95 ± 4.41^†#^ | 14.59 ± 4.54^#^ | 14.23 ± 4.27^#^ | 14.04 ± 4.27^x#^ |
|  | CG | 15.15 ± 3.26 | 14.16 ± 3.57 | 14.48 ± 3.53^#^ | 14.85 ± 3.43^†#^ | 14.91 ± 3.64^#^ | 15.15 ± 3.89^#^ | 15.39 ± 3.33^x#^ |

**Table 7.** Overview of the TMG analysis (energy-restricted (ER) and control group (CG)). * significantly differed to week 1

|  | | **week 1** | **week 3** | **week 5** |
| --- | --- | --- | --- | --- |
| **T_c_ (ms)** | ER | 32.27 ± 7.86 | 34.24 ± 7.33 | 35.31 ± 6.83 |
|  | CG | 31.08 ± 6.70 | 33.12 ± 7.33 | 30.61 ± 5.82 |
| **T_s_ (ms)** | ER | 196.19 ± 64.26 | 178.37 ± 53.89 | 191.37 ± 51.65 |
|  | CG | 152.18 ± 53.25 | 180.11 ± 50.65 | 168.81 ± 48.87 |
| **T_r_ (ms)** | ER | 135.85 ± 68.71 | 108.56 ± 49.62 | 119.20 ± 53.04 |
|  | CG | 81.03 ± 49.34 | 105.10 ± 69.12 | 97.93 ± 66.99 |
| **D_m_ (mm)** | ER | 9.29 ± 2.47 | 10.68 ± 2.58 | 10.20 ± 2.20 |
|  | CG | 9.65 ± 2.56 | 9.86 ± 1.61 | 10.31 ± 2.35 |
| **T_d_ (ms)** | ER | 27.82 ± 3.82 | 28.54 ± 2.46 | 29.10 ± 3.66 |
|  | CG | 26.39 ± 2.29 | 27.19 ± 2.73 | 26.73 ± 2.36 |
| **V_c_ (mm/s)** | ER | 157.67 ± 45.23 | 328.40 ± 114.07* | 161.51 ± 43.15 |
|  | CG | 171.67 ± 53.36 | 313.86 ± 97.99* | 182.52 ± 48.65 |
